# Huperzine A and Its Neuroprotective Molecular Signaling in Alzheimer’s Disease

**DOI:** 10.3390/molecules26216531

**Published:** 2021-10-29

**Authors:** María Jesús Friedli, Nibaldo C. Inestrosa

**Affiliations:** 1 Centro de Excelencia en Biomedicina de Magallanes (CEBIMA), Universidad de Magallanes, Punta Arenas 6210427, Chile; mariajesusfriedli@gmail.com; 2 Departamento de Biología Celular y Molecular, Centro de Envejecimiento y Regeneración (CARE-UC), Facultad de Ciencias Biológicas, Pontificia Universidad Católica de Chile, Santiago 8331150, Chile

**Keywords:** neurodegenerative diseases, huperzine, AChEI, Alzheimer’s disease, therapeutic potential, neuroinflammation

## Abstract

Huperzine A (HupA), an alkaloid found in the club moss *Huperzia serrata*, has been used for centuries in Chinese folk medicine to treat dementia. The effects of this alkaloid have been attributed to its ability to inhibit the cholinergic enzyme acetylcholinesterase (AChE), acting as an acetylcholinesterase inhibitor (AChEI). The biological functions of HupA have been studied both in vitro and in vivo, and its role in neuroprotection appears to be a good therapeutic candidate for Alzheimer´s disease (AD). Here, we summarize the neuroprotective effects of HupA on AD, with an emphasis on its interactions with different molecular signaling avenues, such as the Wnt signaling, the pre- and post-synaptic region mechanisms (synaptotagmin, neuroligins), the amyloid precursor protein (APP) processing, the amyloid-β peptide (Aβ) accumulation, and mitochondrial protection. Our goal is to provide an integrated overview of the molecular mechanisms through which HupA affects AD.

## 1. Introduction

For centuries, *Huperzia serrata* has been used in traditional Chinese medicine under the name “Qian Ceng Ta” as a treatment for schizophrenia, inflammation, swelling, poisoning, pain, and memory loss [1,2,3]. All the properties of *H. serrata* have been intensively studied in China, and most of the biological activity of *H. serrata* appears to be caused by the molecule Huperzine A (HupA). HupA is an unsaturated sesquiterpene alkaloid compound that effectively crosses the blood-brain barrier (BBB), acting as a mixed-competitive, reversible, and selective AChE inhibitor [4,5,6,7] with a half-life of 5 h in the bloodstream, reaching a peak concentration at approximately 60 min in humans, and its t½ has been calculated to be approximately 4–5 h [8].

Its IUPAC name is (1R,9S,13E)-1-amino-13-ethylidene-11-methyl-6-azatricyclo[7.3.1.02,7]trideca-2(7),3,10-trien-5-one. With a molecular weight of 242.32 g/mol [9,10], HupA presents a complex array of structural components: a compact tricyclic structure with a bicyclo [3.1.1] skeleton fused with an α-pyridone ring, an exocyclic ethylidene moiety, and a 3-carbon bridge with an -NH_2_ group. This complex and unique molecular structure accounts for the interesting biological functions and the high stability of HupA [11], plus it results in the presentation of the correct electrostatic field for binding to AChE [12,13]. The three-dimensional structure of AChE complexed with the nootropic alkaloid [-]-HupA was resolved at the Weizmann Institute of Science, Israel [14]. Notably, chemically synthesized [−]-HupA inhibits AChE with similar effectiveness as the natural molecule [10], and [+]-HupA’s AChE inhibitor (AChEI) activity is at least 50-fold less potent than that of the naturally occurring stereoisomer [15].

**Figure 1:**
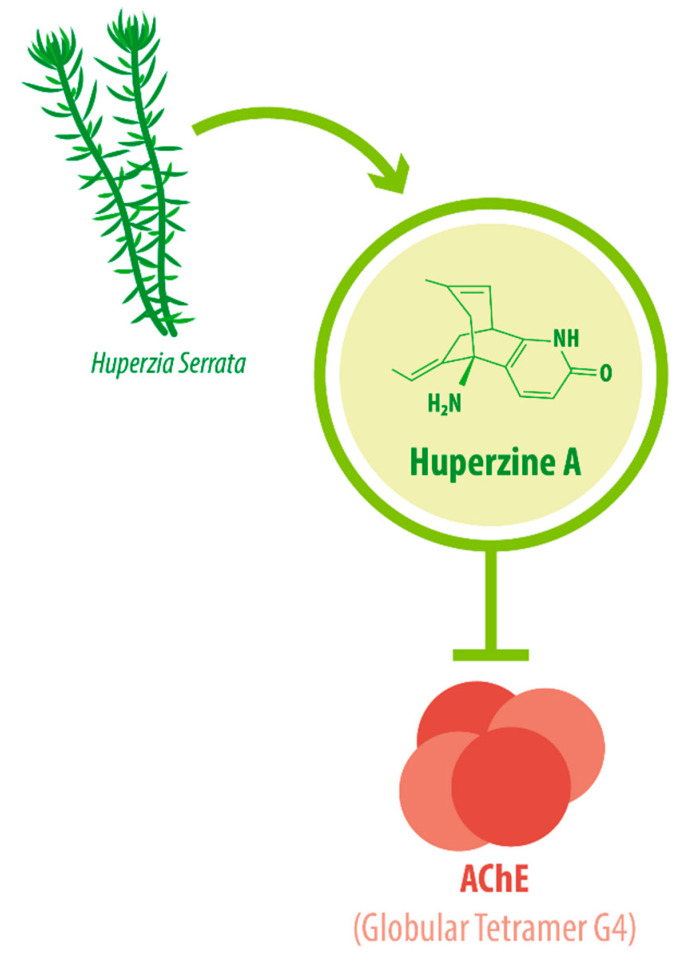
HupA is an acetylcholinesterase inhibitor (AChEI). HupA has a molecular weight of 242.32 g/mol and acts by inhibiting AChE. In the CNS, AChE is present in a tetrameric form anchored to the membrane, or “Globular Tetramer G4” (G4). HupA is the main biological compound in *H. serrata*, which has been used with medicinal purposes in China for centuries. (AChE: acetylcholinesterase; CNS: central nervous system).

The enzyme AChE catalyzes the hydrolysis of the neurotransmitter acetylcholine into choline and acetic acid, regulating the return of cholinergic neurons to a resting state [16]. Most of the cortical AChE activity present in the brain of AD patients is known to be predominantly associated with the amyloid core of senile plaques [17,18]. The globular tetramer (G4) of AChE present in the central nervous system (CNS) [19] (see Figure 1), forms a stable toxic complex with the amyloid-β (Aβ) peptide during its assembly into Aβ filaments and increases the aggregation and neurotoxicity of amyloid fibrils [20,21,22].

**Figure 2:**
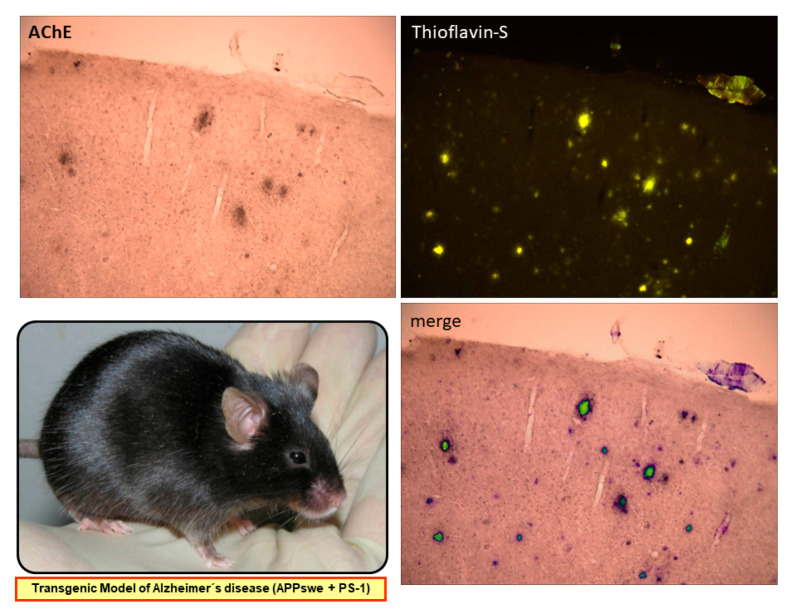
**AChE** interacts with Aβ in amyloid plaques. **Thioflavin-S** is used to stain amyloid plaques. **Merging** the detection of amyloid plaques and of AChE activity confirms colocalization in the brain of an APP/PS1 mouse model of AD (AChE: acetylcholinesterase; APP: amyloid precursor protein; PS1: presenilin 1).

The colocalization of AChE activity with the amyloid plaques in the brain is presented in a double transgenic AD model (APPswe/PS1 mice, see below) (see Figure 2). It is interesting to note that HupA has a higher potency and selectivity of inhibition than commonly prescribed AChEIs galantamine, donepezil, tacrine, and rivastigmine both in vitro and in vivo [9,23].

Due to its potent AChEI activity, HupA is used as a treatment for AD. According to the World Health Organization (WHO), 135.5 million people worldwide will be living with dementia by the year 2050. AD accounts for 65% of all dementia cases [24]. It is a progressive neurological degenerative disorder affecting memory, among other cognitive functions [25,26,27]. Vascular-associated dementia (VaD), on the other hand, is caused by various cerebrovascular diseases, such as cerebral infarction and hemorrhage [28]. These conditions impose great monetary and emotional costs on patients and their caregivers. HupA is approved as the drug of choice for Alzheimer’s disease (AD) treatment in China and as a dietary supplement in the United States [29,30], as intake can improve AD pathogenesis [31].

The biological functions of HupA have been studied both in vitro and in vivo. The present review aims to provide an integrated perspective of the neuroprotective molecular signaling of HupA in dementia, especially in AD.

## 2. HupA within the CNS: Modulation of Critical Pathological Conditions

### 2.1. HupA Mediated Modulation of Amyloid-β PeptideToxicity

One of the hallmarks of AD is the accumulation of Aβ peptide (both oligomers and fibrils) that associate with AChE to form amyloid plaque, producing neuronal cell death [20,21]. The neuroprotective effect of HupA is in part mediated by a dose-dependent reduction in subcellular amyloid-β accumulation in the cortex and hippocampus as shown in a double transgenic mice model carrying the Swedish-mutant amyloid precursor protein (APP) and a deletion in exon 9 of presenilin 1 (PS1) (known as APPswe/PS1 mice) [32], although it is noteworthy that this effect is reportedly absent in TgCRND8 mice [33]. These reductions occur at the protein level through a dose-dependent modulation of the activity of β-site of the APP-cleaving enzyme (BACE1) activity, a reduction in PS1 expression, and a marked increase in *α*-secretase cleavage, promoting the non-amyloidogenic processing of APP [32,34]. An improvement in spontaneous working memory of 68% has been observed in APPswe/PS1 mice after HupA treatment, although HupA does not seem to improve cognition in wild-type (WT) mice [35]. In primary cortical neuron cultures, HupA ameliorates oligomeric Aβ42-induced neurotoxicity by reducing the intracellular accumulation of Aβ42 [36].

HupA modulates the activity of glycogen synthase kinase-3β (GSK-3β), a central molecule of wingless-related integration site (Wnt) signaling cascades [32]. GSK-3β is modulated through inhibitory phosphorylation [37], thus stabilizing the levels of β-catenin in the brain [26,34]. HupA treatment concomitantly decreases the levels of hyperphosphorylated tau protein in both the cortex and the hippocampus, indicating that HupA has effects beyond cholinergic modulation.

At the same time, HupA treatment attenuates the Aβ load in brain mitochondrial homogenates and ameliorates the mitochondrial swelling observed in APPswe/PS1 mice, although it has no effect on WT mice [35]. It has been reported that Aβ accumulation causes mitochondrial dysfunction, resulting in neurotoxicity. HupA treatment restores adenosine triphosphate (ATP) levels and reduces reactive oxygen species (ROS) levels increased by Aβ42 in neurons, and prevents Aβ42-mediated destabilization of the mitochondrial membrane [36]. Notably, HupA treatment reduces Aβ42 accumulation in mitochondria-enriched cellular fractions, but has no significant effects on membrane or cytosol Aβ42 levels, indicating that it exerts a specifically mitochondrial effect [36].

HupA also ameliorates Aβ25–35-induced apoptosis. A proteomic analysis showed that HupA downregulates 29 proteins, including the tumor suppressor protein p53, which has a direct link with apoptosis [37]. In a co-culture system of neural stem cells and microglia exposed to Aβ1–42, HupA treatment partially reduced the secretion of inflammatory factors interleukin-6 (IL-6), tumor necrosis factor-α (TNF-α), and macrophage inflammatory protein-1α (MIP-1α) [37]. HupA also significantly increases the ratio between B-cell lymphoma-2 (Bcl-2) and Bcl-2-like protein 4 (Bax), resulting in improved cell viability. Similarly, HupA directly acts on microglial cells to reduce the expression of cytokines and chemokines [38]. In primary astrocyte cultures, HupA preincubation reduces Aβ1-42-induced cell damage, preventing peaks in the release of p65 subunit of nuclear factor kappa-light-chain-enhancer of activated B cells (NF-kB) [39].

### 2.2. HupA on Dementia

Several studies have evaluated the potential benefits of HupA in human patients. HupA treatment in human subjects suffering from dementia (AD o VaD) shows evidence of improved cognition [4,28,40,41]. It has also been reported that eight weeks of HupA treatment for AD patients improved task switching and alleviated cognitive impairment [42]. The benefits of HupA treatment are apparent when using measurement tools such as Mini-Mental State Exam (MMSE), the AD Assessment Scale-cognitive subscale (ADAS-Cog)) or the ADAS-noncognitive subscale (non-COG) tests. Measuring with tools such as Hastgawa Dementia Scale (HDS) or Wechsler Memory Scale (WMS) tests showed no significant improvement from HupA treatment [24,25]. These results were corroborated in a randomized clinical trial in which HupA treatment in VaD patients significantly improved cognitive function according to the MMSE and Activities of Daily Living (ADL) tests but not according to the Clinical Dementia Rating (CDR) scores [24,28]. However, other trials conclude that HupA treatment could be more beneficial than psychotherapy and conventional medicine for VaD patients [4]. A small trial testing the effects of HupA tablets in AD patients showed that the intake of 0.2 mg of HupA improved cognition and memory in 58% of patients with no severe side effects [43], and a phase II multicenter, 3-arm randomized, double-blind placebo-controlled trial, evaluated as having high methodological quality in later reviews [4], showed significant cognitive enhancement in patients receiving 0.4 mg of HupA twice a day. Remarkably, this dose was well tolerated for 24 weeks even though most AD patients reported being unable to tolerate currently marketed AChEIs for a long period of time [40,44]. A more recent analysis concludes that HupA improves cognition in AD patients, and that its effects are dose and duration-dependent [45].

Some side effects identified in clinical trials are tachycardia, bradycardia, headache, intense dreams, muscle cramps, and arthralgia at high doses. These are well-known cholinergic system-related side effects [8,23]. Nevertheless, these were rated as rare and mild, so HupA remains a well-tolerated drug even in subjects who report intolerance to other AChEIs [4,41]. Some of the described side effects could be mitigated by slow-release formulations or by the synthetic stereoisomer of HupA, which has weaker AChEI activity [11,46].

## 3. Neuroprotective Molecular Signaling of HupA against AD

The neuroprotective effects of HupA treatment result from cholinergic signaling, which increases neurotrophic factor expression, synaptic activity, antagonism of the N-methyl-D-aspartate (NMDA) receptor (NMDAR), modulation of ROS, and neuronal survival [3,9].

Synaptic vesicle release is one of HupA´s neuroprotective mechanisms. During the synaptic vesicle cycle in CNS synapses, synaptic vesicles are filled with neurotransmitters via active transport and form a vesicle pool. Filled vesicles dock at the active zone, where they undergo a priming reaction that makes them competent for Ca^2+^-triggered fusion-pore opening [47]. In this step, synaptotagmins function as Ca^2+^ sensors; in fact, synaptotagmin-1 knockout lacks a synchronized and rapid release of neurotransmitters, showing how relevant their function is [47]. HupA promotes synaptotagmin expression, improving synaptic vesicle exocytosis and neurotransmitter release [48] (see Table 1).

As previously mentioned, HupA inhibits AChE, so, after HupA treatment, acetylcholine will accumulate.Acetylcholine signaling acts mainly through α7nAChRs and α4β2nAChRs, producing an anti-inflammatory response [39]. Acetylcholine signaling modulates the function of neural progenitor cells through the mitogen-activated protein kinases/extracellular signal-regulated kinases (MAPK/ERK) pathway, which is also activated as a response to HupA, exerting neuroprotective functions [49]. The same happens with brain-derived neurotrophic factor (BDNF)/tropomyosin receptor kinase B (TrkB) signaling, which in turn activates the phosphatidylinositol 3-kinase (PI3K)/Akt serine-threonine kinase pathway (see Table 1). Activation of the BDNF/TrkB-dependent PI3K/TrkB/mammalian target of rapamycin (mTOR) signaling pathway suppresses apoptosis and contributes to neuronal survival [50]. The cholinergic increase in BDNF in basal forebrain cholinergic neurons improves the induction and maintenance of long-term potentiation (LTP), synaptic transmission (by acting at pre- and postsynaptic sites), and the presynaptic release of the excitatory neurotransmitter glutamate [51], thus preventing cholinergic neuron loss and preserving cognitive functions [52,53]. Finally, HupA increases the levels of phosphorylated cyclic adenosine monophosphate responsive element-binding protein (CREB) in the hippocampus, which interacts with BDNF in the CREB/BDNF pathway, essential in multiple gene-cognition-environment interactions [54]. One of the main neuroprotective effects of HupA is the amelioration of Aβ accumulation and neurotoxicity through different cholinergic mechanisms [55]. First, through its AChEI activity, HupA disrupts AChE-Aβ interaction, as described in the previous section (see Figure 2) [19,56,57], attenuating p53-mediated neuronal death [37]. HupA exerts a similar action with the postsynaptic cell-adhesion molecule neuroligin 1 (NL1) [48]. NL1 has an extracellular domain with a high degree of similarity to cholinesterases (ChEs) [58,59]. Interaction between NL1 and Aβ oligomers has been reported. The purified extracellular ChE-like domain of NL1 binds directly to Aβ oligomers [60], and Aβ oligomers associate with NL1 at excitatory hippocampal synapses [61]. As with AChE, HupA interacts with the ChE-like domain of NL1 [46], disrupting the Aβ-NL1 interaction and downregulating oligomerization-induced excitotoxicity (see Table 1). Besides disrupting interaction with Aβ oligomers, HupA also alleviates Aβ accumulation. Even though HupA has no effect over the production of the APP, it does modulate its cleavage.

**Table 1:** Molecular targets of HupA’s cholinergic mechanisms in dementia-related pathologies and effects of treatment.

Molecular Target	Effects of HupA Treatment	References
Inhibition of AChE	Reduced neuronal loss Decreased Aβ neurotoxicity]. Attenuated p53-mediated cell death Downregulated NF-kβ signaling Downregulated p65 translocation-related neurodegeneration [	[9,37,39,46,49]
Activation of α7-nAChRs and α4β2-nAChRs	Decreased NF-κB signaling Increased GABAergic transmission Reduced pro-inflammatory cytokines	[31,46,47]
Upregulation of Neurotrophin	Reduced loss of cholinergic neurons	[52]
Activation of BDNF/TrkB	Improved neuronal survival through PI3K/TrkB/mTOR signaling Induced LTP and synaptic transmission	[48,51,52,54]
Upregulation of Bcl-2 and downregulation of Bax	Decreased apoptotic activity	[38]
Upregulation of Synaptotagmin	Improved synaptic vesicle exocytosis	[48]
Interaction with ChE-like domain of Neuroligin-1	Decreased Aβ aggregation	[37,46,60]
Downregulation of activity of GSK-3β	Decreased tau phosphorylation and Aβ accumulation Increased neurogenesis and modulation of synaptic plasticity Increased nonamyloidogenic processing of APP] Downregulated NF-kβ signaling downregulated p65 expression-related neurodegeneration	[39,46,48,54]

(AChE: acetylcholinesterase; nAChRs: nicotinic acetylcholine receptors; Bcl-2: B-cell lymphoma-2; Bax: Bcl-2-like protein 4; Gsk-3β: glycogen synthase kinase-3β: mTOR: mammalian target of rapamycin; BDNF: brain-derived neurotrophic factor; TrkB: tropomyosin receptor kinase B).

The APP is cleaved by one of two possible pathways. In the α-secretase nonamyloidogenic pathway, the APP is cleaved by an α-secretase such as disintegrin and metalloproteinase domain-containing protein 10 (ADAM10), producing the soluble ectodomain of APP (sAPPα) and a membrane-tethered intracellular C-terminal fragment (C83). Further processing by a γ-secretase cleaves the C83, yielding a 3-kDa peptide (P3) and an APP intracellular domain (AICD) [62]. P3 and AICD are both neurotrophic and neuroprotective [26]. On the other hand, in the β/γ-secretase amyloidogenic pathway, the cleavage is made by BACE1, and it yields the slightly shorter soluble APPβ fragment (sAPPβ) and a C99 fragment. Further processing by a γ-secretase cleaves C99 and releases the AICD and the Aβ peptide, a hallmark of AD pathology [62].

**Figure 3:**
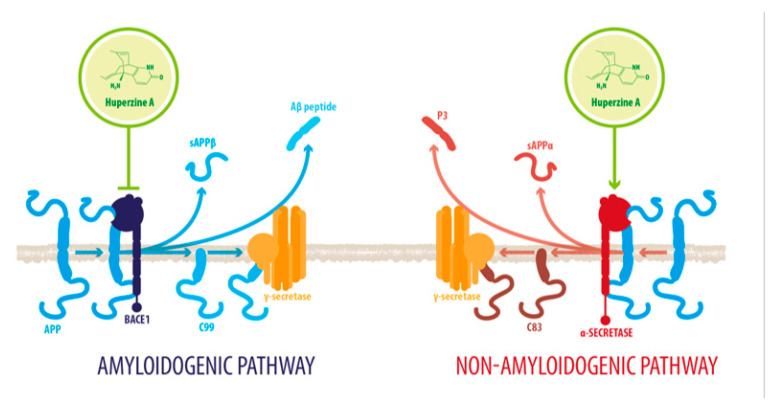
HupA regulates APP processing. HupA dose-dependently increases the activity of the *nonamyloidogenic* pathway through the cleavage of amyloid precursor protein (APP) by an α-secretase, producing soluble α-APP fragments, P3 peptide and a C83 fragment. These are both neurotrophic and neuroprotective and are upregulated after HupA treatment. HupA also reduces the levels of BACE1, a β/γ-secretase that produces soluble β-APP fragments in what is known as the *amyloidogenic* pathway, in addition to the C99 fragment that is cleaved to form the Aβ peptide. These products trigger apoptotic responses, such as neuronal membrane blebbing and cell shrinkage, leading to cell death (APP: amyloid precursor protein; BACE1: beta-site amyloid precursor protein cleaving enzyme 1; sAPPβ: soluble amyloid precursor protein-β; sAPPα: soluble amyloid precursor protein-α).

HupA increases the expression of the sAPPa and C83 fragments [26,32], and it dose-dependently restores the constitutive expression of ADAM10, while downregulating the membrane translocation of BACE1, which hinders BACE1-APP association (see Figure 3).

**Figure 4:**
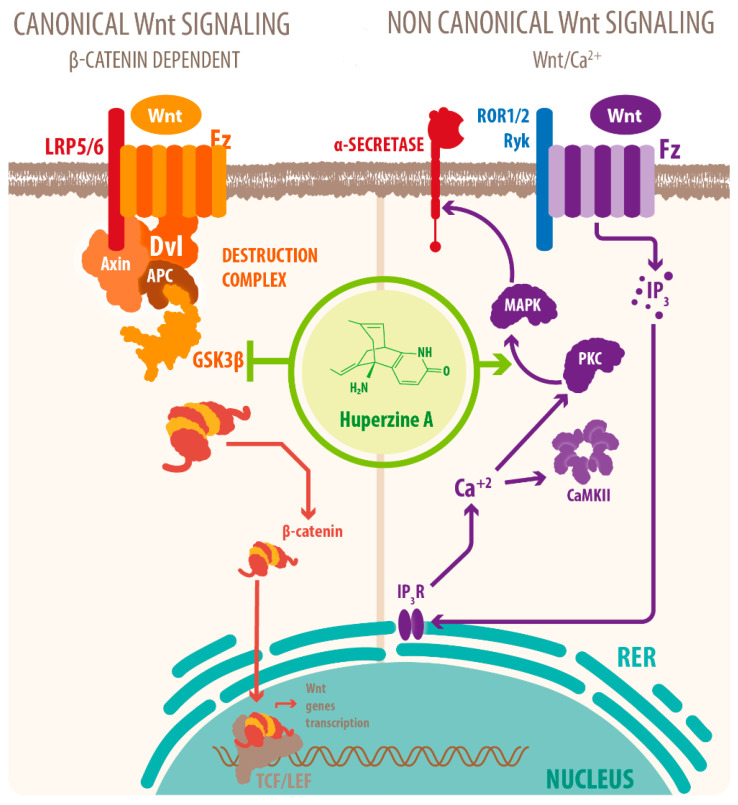
HupA affects Wnt signaling. HupA affects both canonical and noncanonical Wnt signaling. Regarding the canonical signaling, HupA modulates the Wnt/β-catenin pathway by downregulating GSK-3β activity. HupA increases the phosphorylation levels of GSK-3α/β, which stabilizes β-catenin and upregulates expression of its target genes, related to synaptic plasticity and neuronal survival, while preventing tau hyperphosphorylation and Aβ neurotoxicity. HupA also interacts with the noncanonical pathway via PKC/MAPK signaling. PKC activation also inhibits GSK-3β activity and plays a role in upregulating the expression of the α-secretase, preventing Aβ accumulation and neuronal apoptosis (Wnt: wingless-related integration site; GSK3: glycogen synthase kinase 3; PKC: protein kinase C; MAPK: mitogen-activated protein kinase).

Activation of mAChRs and protein kinase C (PKC)/MAPK pathways have been proposed as a mechanism for this effect [15,23,46], as well as HupA mediated increase of phosphorylation of ERK1/2, that can also regulate ADAM10 transcription. Another mechanism for the cholinergic modulation of APP processing is the activation of Wnt signaling. Wnt signaling plays a crucial role in presynaptic assembly, postsynaptic differentiation at vertebrate peripheral neuromuscular synapses, stem cell differentiation, and the development and maintenance of the CNS [63,64,65]. Recent evidence suggests that it also regulates vascular stability, BBB integrity, and inflammation [66]. In the canonical Wnt pathway or Wnt/β-catenin pathway, Wnt ligands bind to receptors of the Frizzled (FZD) and low-density lipoprotein-related protein (LRP) families on the cell surface and activate several cytoplasmic relay components, including GSK-3β and adenomatous polyposis coli (APC), finally signaling to β-catenin. β-catenin enters the nucleus and forms a complex with lymphoid enhancer-binding factor 1 (LEF1) to activate the transcription of Wnt target genes. Wnt signaling pathways that are independent of β-catenin are referred to as non-canonical pathways, which include the planar cell polarity (PCP) pathway, also known as the Wnt/Jun N-terminal kinase (JNK) pathway, and the Wnt/ Ca^2+^ pathway [63] (see Figure 4).

Inhibition of Wnt signaling causes cognitive deficits, increases tau phosphorylation, increases Aβ1-42 peptide concentrations and the Aβ42/Aβ40 ratio in the hippocampus, and decreases Aβ1-42 concentrations in the cerebrospinal fluid, indicating insufficient clearance across the BBB [18]. Thus, activation of Wnt signaling by HupA ameliorates Aβ burden and cognitive deficits [23,25,32].

Wnt signaling also modulates the nonamyloidogenic processing of APP, as transcriptional activation of Wnt target genes by β-catenin translocation directs APP processing towards ADAM10 while repressing BACE1 transcription, thus linking Wnt signalling and Aβ accumulation [67], as mentioned earlier. GSK-3 is one of Wnt´s cytoplasmic relay components, and its increased activation causes increased phosphorylation of β-catenin, which inhibits canonical Wnt signaling [63], and AD hallmarks such as excessive tau protein phosphorylation and Aβ generation. In addition, it has been shown that Aβ impairs PI3K/Akt signaling, leading to the activation of GSK-3β. This creates a feedback loop that induces tau hyperphosphorylation, neurofibrillary tangle formation, synaptotoxicity, synaptic loss, and eventually neuronal death [64]. However, as was previously mentioned, HupA increases PKC signaling, and PKC inhibits GSK-3β activity, preventing tau phosphorylation and Aβ production (see Figure 4) [32]. Through the activation of Wnt and PKC signaling, HupA treatment rescues the nonamyloidogenic processing of APP, indicating that these pathways might be central to HupA´s neuroprotective effects against Aβ burden [32] (see Table 1). Furthermore, through these mechanisms, HupA protects the presynaptic structure and synaptic plasticity [26,32,61,68] (see Table 1).

**Figure 5:**
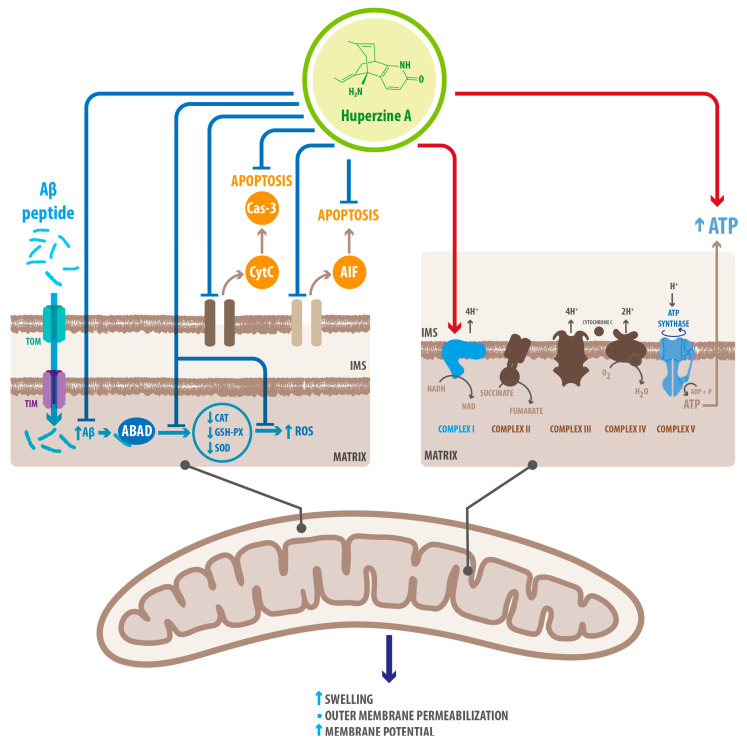
HupA rescues mitochondrial integrity after exposure to Aβ. Aβ crosses the mitochondrial double membrane and aggregates in the matrix. Mitochondrial perturbations include changes in morphology, compromised TCA cycle and reduced CytoC oxidase activity. Cell death is initiated by releasing proteins from intermembrane and intracristal spaces which activates caspases and AIF (caspase-independent apoptosis pathway). Outer membrane permeabilization leads to mitochondrial metabolic failure with subsequent mitochondrial swelling, ROS accumulation and neuronal death. HupA treatment prevents Aβ accumulation in the matrix as well as Aβ interaction with mitochondrial proteins. HupA prevents the release of CytoC and subsequent apoptosis. It also rescues overall membrane stability, thus protecting mitochondrial enzymatic activity, membrane potential, and ATP formation. HupA also enriches proteins involved in oxidative phosphorylation, improving mitochondrial respiration (ABAD: amyloid beta-binding alcohol dehydrogenase; CAT: catalase; GSH-PX: glutathione peroxidase; SOD: superoxide dismutase; ROS: reactive oxygen species; IMS: intermembrane space; CytoC: cytochrome C; Cas-3: caspase-3; AIF: apoptosis inducing factor).

HupA also acts via non-cholinergic mechanisms, targeting mitochondrial structure and function as well as Fe^2+^homeostasis. Mitochondrial perturbations have been described in AD pathology, including changes in morphology, a compromised tricarboxylic acid (TCA) cycle, reduced cytochrome C (CytoC) oxidase activity, and oxidative stress [69]. Aβ can accumulate in the mitochondria, interacting with the mitochondrial protein Aβ-binding alcohol dehydrogenase (ABAD), causing perturbations that lead to neurotoxicity and apoptosis. Aβ accumulation and mitochondrial dysfunction synergize to activate neurodegenerative pathways involved in cognitive loss [52,53]. Mitochondria initiate apoptosis by releasing proteins such as CytoC from intermembrane and intracristal spaces, followed by the activation of caspases and apoptosis inducing factor (AIF) (a caspase-independent apoptosis pathway). Outer membrane permeabilization leadsto mitochondrial metabolic failure with subsequent mitochondrial swelling and neuronal death [69]. In turn, APP overexpression may affect mitochondrial function by disrupting NIPSNAP1 protein localization in the mitochondrial matrix through direct interaction, altering the binding of the dihydrolipoyl transacylase and dihydrolipoyl transacetylase components of the branched chain ketoacids complex and pyruvate dehydrogenase complex in the mitochondrial interior membrane [37] (see Figure 5). HupA inhibits the cytoplasmic translocation of CytoC and caspase-3 cleavage [69], attenuating apoptotic-inducing signals (see Table 2), and possibly prevents APP-induced mitochondrial dysfunction by reversing the disruption of NIPSNAP1 protein localization in the mitochondrial matrix [37]. HupA also upregulates glutathione peroxidase (GSH-PX), superoxide dismutase (SOD), and catalase (CAT), thus preventing oxidative stress [70]. HupA mediated neuroprotection possibly involves restoration of an intact double membrane, which restores the membrane potential and ATP production, meaning preserved energy homeostasis [35,52] (see Table 2). Proteomic analysis confirms that HupA increases the expression of proteins involved in oxidative phosphorylation [37].

**Figure 6:**
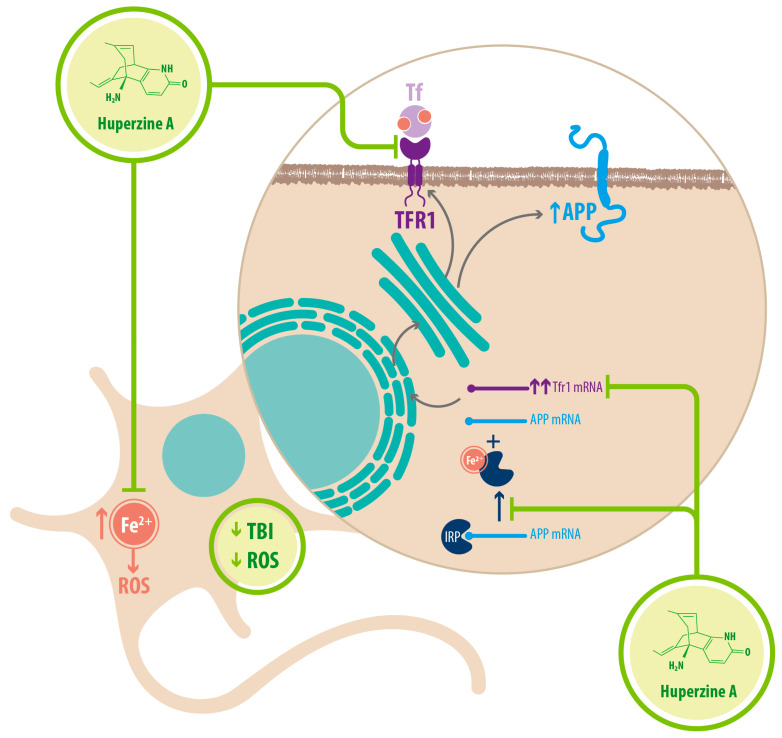
HupA regulates Fe^2+^ homeostasis. HupA prevents the accumulation of Fe^2+^ inside the neurons. It might act as a chelator, while also downregulating the expression of TFR1 and preventing Fe^2+^ uptake into the cell. By reducing Fe^2+^ concentration inside the neuron, HupA reduces APP translation. Fe^2+^ binds the IRP, releasing it from APP mRNA which can then get translated. In the presence of HupA, Fe^2+^concentration drops, and less IRP is released, meaning less APP is translated. HupA indirectly reduces ROS through all these mechanisms (TFR1: transferrin receptor 1; Tf: transferrin; TBI: transferrin bound iron; ROS: reactive oxygen species; APP: amyloid precursor protein; IRP: iron regulatory protein).

Regarding Fe^2+^ homeostasis, it has been reported that AD mice models have elevated levels of Fe^2+^ in the cortex and hippocampus compared with WT mice. Accordingly, the expression of transferrin receptor protein 1 (TFR1) is upregulated in APPswe/PS1dE9 mice, which explains the increase in Fe^2+^ uptake [34]. Fe^2+^-mediated neurodegeneration might originate in the downregulation of α-secretase activity, inhibiting the nonamyloidogenic pathway, with concomitant increase of APP expression due to a functional iron-responsive element (IRE) that has been identified in the APP promoter, which would explain why APP levels increase after Fe^2+^ treatment and decrease after chelator treatment. An alternative explanation could be that the translational expression of APP could be directed via release of a classic repressor interaction between APP mRNA and iron regulatory protein-1 (IRP-1) [34]. HupA might act directly as an Fe^2+^ chelator, reducing the capacity of IRP-1 to induce APP translation [34]. HupA also downregulates TFR1 expression in mice in vivo, which reduces the uptake of transferrin-bound iron (TBI) in neurons [34] (see Figure 6) (see Table 2).

Finally, it is noteworthy that HupA reduces proinflammatory cytokines expression through activation of nAChRs. Activating the cholinergic anti-inflammatory pathway, the efferent arc of the inflammatory reflex in the CNS, results in suppressed inflammatory cytokine expression and systemic inflammatory responses. The activation of α7nAChR downregulates macrophages, microglia, and astrocytes activation inhibiting the production of proinflammatory cytokines while anti-inflammatory cytokine expression is not altered [10,46,71]. The cholinergic anti-inflammatory effects of HupA downregulates IL-1β, IL-6, and TNF-α secretion and NF-kB signaling, attenuating neuroinflammatory response [72].

## 4. Concluding Remarks

HupA has therapeutic benefits for the treatment of AD, but the understanding of molecular interactions involved in the recovery of neuronal function after HupA treatment is still incomplete. What is known is that AChEI activity is crucial through the regulation of Aβ peptide accumulation by activation of α-secretase cleavage and down-regulation of β/γ-secretase, enhancing BDNF/TrkB signaling as well as PI3K/Akt and PI3K/TrkB/mTOR pathways. Concomitant reduction of IL-1β, IL-6, TNF-α, and NF-kB signaling preserves neuronal function. Modulation of Wnt signaling through HupA treatment might be the most promising therapeutic target, as Wnt is involved in neuronal survival and in synaptic plasticity. There are also non-cholinergic neuroprotective effects such as the preservation of mitochondrial structure and function under Aβ insult, as well as the recovery of Fe^2+^ homeostasis in the brain. Further research and development of HupA as a common treatment might alleviate the burden related to highly prevalent conditions that are costly in terms of public health policies and devastating to those who bear them and their caretakers.

## Figures and Tables

**Table 2:** Molecular targets of HupA’s noncholinergic mechanisms in dementia-related pathologies and effects of treatment.

Mechanism	Molecular Target	Effects of HupA Treatment	References
Mitochondrial protection	Upregulation of oxidative phosphorylation proteins	Recovered energetic homeostasis	[52]
	Inhibition of cytoplasmic translocation of CytoC	Prevented mitochondria-mediated apoptosis	[69]
	Downregulation of AIF	Prevented cas3-independent apoptosis	[52]
	Inhibition of Caspase-3 cleavage	Prevented cas-3 dependent apoptosis	[52]
	Upregulation of GSH-PX SOD CAT	Decreased ROS accumulation Decreased oxidative stress	[52]
Fe^2+^ homeostasis	Chelator/ Reducer of Fe^2+^	Increased nonamyloidogenic processing of APP Downregulated expression of APP through IRP-1	[34]
	Downregulation of TFR1	Decreased Fe^2+^ uptake in neurons	[34]

(CytoC: cytochrome C; Cas-3: caspase-3; AIF: apoptosis inducing factor; CAT: catalase; GSH-PX: glutathione peroxidase; SOD: superoxide dismutase; TFR1: transferrin receptor 1; ROS: reactive oxygen species; APP: amyloid precursor protein; IRP: iron regulatory protein).

## Data Availability

The data presented in this study are available within the article (tables and figures).

## Abbreviations

AChE	Acetylcholinesterase
AChEI	Acetylcholinesterase Inhibitor
AD	Alzheimer´s Disease
ADAM10	Disintegrin and Metalloproteinase Domain-containing Protein 10
AIF	Apoptosis Inducing Factor
APP	Amyloid Precursor Protein
ATP	Adenosine Triphosphate
Aβ	Amyloid- β peptide
BACE1	β-site of the APP-cleaving enzyme
Bax	Bcl-2-like protein 4
BBB	Blood Brain Barrier
Bcl-2	Bcl-2 B-cell lymphoma-2
BDNF	Brain-Derived Neurotrophic Factor
CAT	Catalase
CNS	Central Nervous System
CREB	Cyclic Adenosine Monophosphate Responsive Element Binding Protein
CytoC	Cytochrome C
GSH-PX	Glutathione Peroxidase
GSK-3β	Glycogen Synthase Kinase-3β
HupA	Huperzine A
IL-6	Interleukin-6
IRE	Iron-Responsive Element
IRP-1	Iron Regulatory Protein-1
MAPK/ERK	Mitogen-activated Protein Kinases/Extracellular Signal-regulated Kinases
MIP-1α	Macrophage Inflammatory Protein-1α
mTOR	Mammalian Target of Rapamycin
NF-kB	Nuclear Factor Kappa-light-chain-enhancer of Activated B cells
NL1	Neuroligin 1
NMDA	N-methyl-D-aspartate
NMDAR	N-methyl-D-aspartate Receptor
PI3K	Phosphatidylinositol 3-Kinase
PKC	Protein Kinase C
PS1	Presenilin 1
ROS	Reactive Oxygen Species
SOD	Superoxide Dismutase
TBI	Transferrin-Bound Iron
TFR1	Transferrin Receptor Protein 1
TNF-α	Tumor Necrosis Factor-α
TrkB	Tropomyosin receptor kinase B
VaD	Vascular-associated Dementia
Wnt	Wingless-related Integration Site
WT	Wild-type

## References

[1] Howes M.R., Fang R., Houghton P.J. (2017). Effect of Chinese Herbal Medicine on Alzheimer’s Disease. Int. Rev. Neurobiol..

[2] Murphy R.A., Sarpong R. (2014). Heathcock-inspired strategies for the synthesis of fawcettimine-type Lycopodium alkaloids. Chemistry.

[3] Wu T.Y., Chen C.P., Jinn T.R. (2011). Traditional Chinese medicines and Alzheimer’s disease. Taiwan J. Obstet. Gynecol..

[4] Yang G., Wang Y., Tian J., Liu J.P. (2013). Huperzine A for Alzheimer’s disease: A systematic review and meta-analysis of randomized clinical trials. PLoS ONE.

[5] Nett R.S., Dho Y., Low Y., Sattely E.S. (2021). A metabolic regulon reveals early and late acting enzymes in neuroactive Lycopodium alkaloid biosynthesis. Proc. Natl. Acad. Sci. USA.

[6] Callizot N., Campanari M.L., Rouvière L., Jacquemot G., Henriques A., Garayev E., Poindron P. (2021). Huperzia serrata Extract ‘NSP01’ with Neuroprotective Effects-Potential Synergies of Huperzine A and Polyphenols. Front. Pharmacol..

[7] Kong Y.R., Tay K.C., Su Y.X., Wong C.K., Tan W.N., Khaw K.Y. (2021). Potential of Naturally Derived Alkaloids as Multi-Targeted Therapeutic Agents for Neurodegenerative Diseases. Molecules.

[8] De La Garza R., Verrico C.D., Newton T.F., Mahoney J.J., Thompson-Lake D.G. (2015). Safety and Preliminary Efficacy of the Acetylcholinesterase Inhibitor Huperzine A as a Treatment for Cocaine Use Disorder. Int. J. Neuropsychopharmacol..

[9] Ohba T., Yoshino Y., Ishisaka M., Abe N., Tsuruma K., Shimazawa M., Oyama M., Tabira T., Hara H. (2015). Japanese Huperzia serrata extract and the constituent, huperzine A, ameliorate the scopolamine-induced cognitive impairment in mice. Biosci. Biotechnol. Biochem..

[10] Damar U., Gersner R., Johnstone J.T., Schachter S., Rotenberg A. (2016). Huperzine A as a neuroprotective and antiepileptic drug: A review of preclinical research. Expert Rev. Neurother..

[11] Ferreira A., Rodrigues M., Fortuna A., Falcão A., Alves G. (2016). Huperzine A from Huperzia serrata: A review of its sources, chemistry, pharmacology and toxicology. Phytochem. Rev..

[12] Ashani Y., Grunwald J., Kronman C., Velan B., Shafferman A. (1994). Role of tyrosine 337 in the binding of huperzine A to the active site of human acetylcholinesterase. Mol. Pharmacol..

[13] Pang Y.P., Kozikowski A.P. (1994). Prediction of the binding sites of huperzine A in acetylcholinesterase by docking studies. J. Comput. Aided Mol. Des..

[14] Raves M.L., Harel M., Pang Y.P., Silman I., Kozikowski A.P., Sussman J.L. (1997). Structure of acetylcholinesterase complexed with the nootropic alkaloid, (-)-huperzine A. Nat. Struct. Biol..

[15] Tun M.K., Herzon S.B. (2012). The pharmacology and therapeutic potential of (-)-huperzine A. Int. J. Exp. Pharmacol..

[16] Colović M.B., Krstić D.Z., Lazarević-Pašti T.D., Bondžić A.M., Vasić V.M. (2013). Acetylcholinesterase inhibitors: Pharmacology and toxicology. Curr. Neuropharmacol..

[17] Geula C., Mesulam M.M., Saroff D.M., Wu C.K. (1998). Relationship between plaques, tangles, and loss of cortical cholinergic fibers in Alzheimer disease. J. Neuropathol. Exp. Neurol..

[18] Reyes A.E., Chacón M.A., Dinamarca M.C., Cerpa W., Morgan C., Inestrosa N.C. (2004). Acetylcholinesterase-Abeta complexes are more toxic than Abeta fibrils in rat hippocampus: Effect on rat beta-amyloid aggregation, laminin expression, reactive astrocytosis, and neuronal cell loss. Am. J. Pathol..

[19] Fuentes M.E., Inestrosa N.C. (1988). Characterization of a tetrameric G4 form of acetylcholinesterase from bovine brain: A comparison with the dimeric G2 form of the electric organ. Mol. Cell. Biochem..

[20] Inestrosa N.C., Alvarez A., Pérez C.A., Moreno R.D., Vicente M., Linker C., Casanueva O.I., Soto C., Garrido J. (1996). Acetylcholinesterase accelerates assembly of amyloid-b-peptides into Alzheimer’s fibrils: Possible role of the peripheral site of the enzyme. Neuron.

[21] Alvarez A., Alarcón R., Opazo C., Campos E.O., Muñoz F.J., Calderón F.H., Dajas F., Gentry M.K., Doctor B.P., De Mello F.G. (1998). Stable Complexes Involving Acetylcholinesterase and Amyloid-β Peptide Change the Biochemical Properties of the Enzyme and Increase the Neurotoxicity of Alzheimer’s Fibrils. J. Neurosci..

[22] Bartolini M., Bertucci C., Cavrini V., Andrisano V. (2003). β-Amyloid aggregation induced by human acetylcholinesterase: Inhibition studies. Biochem. Pharmacol..

[23] Zhang H.Y. (2012). New insights into huperzine A for the treatment of Alzheimer’s disease. Acta Pharmacol. Sin..

[24] Zhou X., Cui G., Tseng H.H., Lee S.M., Leung G.P., Chan S.W., Kwan Y.W., Hoi M.P. (2016). Vascular Contributions to Cognitive Impairment and Treatments with Traditional Chinese Medicine. Evid. Based Complementary Altern. Med..

[25] Ha G.T., Wong R.K., Zhang Y. (2011). Huperzine a as potential treatment of Alzheimer’s disease: An assessment on chemistry, pharmacology, and clinical studies. Chem. Biodivers..

[26] Tapia-Rojas C., Inestrosa N.C. (2018). Wnt signaling loss accelerates the appearance of neuropathological hallmarks of Alzheimer’s disease in J20-APP transgenic and wild-type mice. J. Neurochem..

[27] Rahman M., Bajgai J., Fadriquela A., Sharma S., Trinh T.T., Akter R., Lee K.J. (2021). Therapeutic Potential of Natural Products in Treating Neurodegenerative Disorders and Their Future Prospects and Challenges. Molecules.

[28] Xu Z.Q., Liang X.M., Juan W., Zhang Y.F., Zhu C.X., Jiang X.J. (2012). Treatment with Huperzine A improves cognition in vascular dementia patients. Cell Biochem. Biophys..

[29] Yang Y., Wang Z., Wu J., Chen Y. (2016). Chemical Constituents of Plants from the Genus Phlegmariurus. Chem. Biodivers..

[30] Orhan I.E., Orhan G., Gurkas E. (2011). An overview on natural cholinesterase inhibitors—A multi-targeted drug class-and their mass production. Mini Rev. Med. Chem..

[31] Chauhan P.S., Yadav D. (2021). Dietary Nutrients and Prevention of Alzheimer’s disease. CNS Neurol. Disord. Drug Targets.

[32] Wang C.Y., Zheng W., Wang T., Xie J.W., Wang S.L., Zhao B.L., Teng W.P., Wang Z.Y. (2011). Huperzine A activates Wnt/β-catenin signaling and enhances the nonamyloidogenic pathway in an Alzheimer transgenic mouse model. Neuropsychopharmacology.

[33] Yuan Q., Lin Z.X., Wu W., Albert W.N., Zee B.C.Y. (2020). Huperzine A in treatment of amyloid-β-associated neuropathology in a mouse model of Alzheimer disease: Abridged secondary publication. Hong Kong Med. J..

[34] Huang X.T., Qian Z.M., He X., Gong Q., Wu K.C., Jiang L.R., Lu L.N., Zhu Z.J., Zhang H.Y., Yung W.H. (2014). Reducing iron in the brain: A novel pharmacologic mechanism of huperzine A in the treatment of Alzheimer’s disease. Neurobiol. Aging.

[35] Yang L., Ye C.Y., Huang X.T., Tang X.C., Zhang H.Y. (2012). Decreased accumulation of subcellular amyloid-β with improved mitochondrial function mediates the neuroprotective effect of huperzine A. J. Alzheimer’s Dis..

[36] Lei Y., Yang L., Ye C.Y., Qin M.Y., Yang H.Y., Jiang H.L., Tang X.C., Zhang H.Y. (2015). Involvement of Intracellular and Mitochondrial Aβ in the Ameliorative Effects of Huperzine A against Oligomeric Aβ42-Induced Injury in Primary Rat Neurons. PLoS ONE.

[37] Tao Y., Fang L., Yang Y., Jiang H., Yang H., Zhang H., Zhou H. (2013). Quantitative proteomic analysis reveals the neuroprotective effects of huperzine A for amyloid beta treated neuroblastoma N2a cells. Proteomics.

[38] Zhu N., Lin J., Wang K., Wei M., Chen Q., Wang Y. (2015). Huperzine A protects neural stem cells against Aβ-induced apoptosis in a neural stem cells and microglia co-culture system. Int. J. Clin. Exp. Pathol..

[39] Xie L., Jiang C., Wang Z., Yi X., Gong Y., Chen Y., Fu Y. (2016). Effect of Huperzine A on Aβ-induced p65 of astrocyte in vitro. Biosci. Biotechnol. Biochem..

[40] Rafii M.S., Walsh S., Little J.T., Behan K., Reynolds B., Ward C., Jin S., Thomas R., Aisen P.S. (2011). A phase II trial of huperzine A in mild to moderate Alzheimer disease. Neurology.

[41] Xing S.H., Zhu C.X., Zhang R., An L. (2014). Huperzine a in the treatment of Alzheimer’s disease and vascular dementia: A meta-analysis. Evid. Based Complementary Altern. Med..

[42] Gul A., Bakht J., Mehmood F. (2019). Huperzine-A response to cognitive impairment and task switching deficits in patients with Alzheimer’s disease. J. Chin. Med Assoc..

[43] Xu S.S., Gao Z.X., Weng Z., Du Z.M., Xu W.A., Yang J.S., Zhang M.L., Tong Z.H., Fang Y.S., Chai X.S. (1995). Efficacy of tablet huperzine-A on memory, cognition, and behavior in Alzheimer’s disease. Zhongguo Yao Li Xue Bao.

[44] Tsai S. (2019). Huperzine-A, a versatile herb, for the treatment of Alzheimer’s disease. Crit. Care Med..

[45] Ghassab-Abdollahi N., Mobasseri K., Dehghani Ahmadabad A., Nadrian H., Mirghafourvand M. (2021). The effects of Huperzine A on dementia and mild cognitive impairment: An overview of systematic reviews. Phytother. Res..

[46] Damar U., Gersner R., Johnstone J.T., Schachter S., Rotenberg A. (2017). Huperzine A: A promising anticonvulsant, disease modifying, and memory enhancing treatment option in Alzheimer’s disease. Med. Hypotheses.

[47] Südhof T.C. (2013). Neurotransmitter release: The last millisecond in the life of a synaptic vesicle. Neuron.

[48] Wang Y., Tang X.C., Zhang H.Y. (2012). Huperzine A alleviates synaptic deficits and modulates amyloidogenic and nonamyloidogenic pathways in APPswe/PS1dE9 transgenic mice. J. Neurosci. Res..

[49] Ma T., Gong K., Yan Y., Zhang L., Tang P., Zhang X., Gong Y. (2013). Huperzine A promotes hippocampal neurogenesis in vitro and in vivo. Brain Res..

[50] Mao X.Y., Zhou H.H., Li X., Liu Z.Q. (2016). Huperzine A Alleviates Oxidative Glutamate Toxicity in Hippocampal HT22 Cells via Activating BDNF/TrkB-Dependent PI3K/Akt/mTOR Signaling Pathway. Cell. Mol. Neurobiol..

[51] Kerr F., Bjedov I., Sofola-Adesakin O. (2018). Molecular Mechanisms of Lithium Action: Switching the Light on Multiple Targets for Dementia Using Animal Models. Front. Mol. Neurosci..

[52] Qian Z.M., Ke Y. (2014). Huperzine A: Is it an Effective Disease-Modifying Drug for Alzheimer’s Disease?. Front. Aging Neurosci..

[53] Rahman M.H., Bajgai J., Fadriquela A., Sharma S., Trinh Thi T., Akter R., Goh S.H., Kim C.-S., Lee K.-J. (2021). Redox Effects of Molecular Hydrogen and Its Therapeutic Efficacy in the Treatment of Neurodegenerative Diseases. Processes.

[54] Du Y., Liang H., Zhang L., Fu F. (2017). Administration of Huperzine A exerts antidepressant-like activity in a rat model of post-stroke depression. Pharmacol. Biochem. Behav..

[55] Mak S., Li W., Fu H., Luo J., Cui W., Hu S., Pang Y., Carlier P.R., Tsim K.W., Pi R. (2021). Promising tacrine/huperzine A-based dimeric acetylcholinesterase inhibitors for neurodegenerative disorders: From relieving symptoms to modifying diseases through multitarget. J. Neurochem..

[56] Carvajal F.J., Inestrosa N.C. (2011). Interactions of AChE with Aβ Aggregates in Alzheimer’s Brain: Therapeutic Relevance of IDN 5706. Front. Mol. Neurosci..

[57] Rees T., Hammond P.I., Soreq H., Younkin S., Brimijoin S. (2003). Acetylcholinesterase promotes beta-amyloid plaques in cerebral cortex. Neurobiol. Aging.

[58] Leone P., Comoletti D., Taylor P., Bourne Y., Marchot P. (2010). Structure-function relationships of the alpha/beta-hydrolase fold domain of neuroligin: A comparison with acetylcholinesterase. Chem. Biol. Interact..

[59] Scholl F.G., Scheiffele P. (2003). Making connections: Cholinesterase-domain proteins in the CNS. Trends Neurosci..

[60] Dinamarca M.C., Sagal J.P., Quintanilla R.A., Godoy J.A., Arrázola M.S., Inestrosa N.C. (2010). Amyloid-beta-Acetylcholinesterase complexes potentiate neurodegenerative changes induced by the Abeta peptide. Implications for the pathogenesis of Alzheimer’s disease. Mol. Neurodegener..

[61] Dinamarca M.C., Di Luca M., Godoy J.A., Inestrosa N.C. (2015). The soluble extracellular fragment of neuroligin-1 targets Aβ oligomers to the postsynaptic region of excitatory synapses. Biochem. Biophys. Res. Commun..

[62] Nhan H.S., Chiang K., Koo E.H. (2015). The multifaceted nature of amyloid precursor protein and its proteolytic fragments: Friends and foes. Acta Neuropathol..

[63] Inestrosa N.C., Arenas E. (2010). Emerging roles of Wnts in the adult nervous system. Nat. Rev. Neurosci..

[64] Oliva C.A., Montecinos-Oliva C., Inestrosa N.C. (2018). Wnt Signaling in the Central Nervous System: New Insights in Health and Disease. Prog. Mol. Biol. Transl. Sci..

[65] Varela-Nallar L., Inestrosa N.C. (2013). Wnt signaling in the regulation of adult hippocampal neurogenesis. Front. Cell. Neurosci..

[66] Menet R., Lecordier S., ElAli A. (2020). Wnt Pathway: An Emerging Player in Vascular and Traumatic Mediated Brain Injuries. Front. Physiol..

[67] Tapia-Rojas C., Burgos P.V., Inestrosa N.C. (2016). Inhibition of Wnt signaling induces amyloidogenic processing of amyloid precursor protein and the production and aggregation of Amyloid-β (Aβ)(42) peptides. J. Neurochem..

[68] Inestrosa N.C., Varela-Nallar L. (2014). Wnt signaling in the nervous system and in Alzheimer’s disease. J. Mol. Cell Biol..

[69] Lu H., Jiang M., Lu L., Zheng G., Dong Q. (2015). Ultrastructural mitochondria changes in perihematomal brain and neuroprotective effects of Huperzine A after acute intracerebral hemorrhage. Neuropsychiatr. Dis. Treat..

[70] Yang X., Wei H.M., Hu G.Y., Zhao J., Long L.-N., Li C.-J., Zhao Z.-J., Zeng H.-K., Nie H. (2020). Combining antioxidant astaxantin and cholinesterase inhibitor huperzine A boosts neuroprotection. Mol. Med. Rep..

[71] Báez-Pagán C.A., Delgado-Vélez M., Lasalde-Dominicci J.A. (2015). Activation of the Macrophage α7 Nicotinic Acetylcholine Receptor and Control of Inflammation. J. Neuroimmune Pharmacol..

[72] Donat C.K., Scott G., Gentleman S.M., Sastre M. (2017). Microglial Activation in Traumatic Brain Injury. Front. Aging Neurosci..

